# Supplementary material for: Multi‐dataset identification of innovative feature genes and molecular mechanisms in keratoconus
Source: J Cell Mol Med. 2024 Sep 19;28(18):e70079. doi: 10.1111/jcmm.70079 (PMC11412914; doi:10.1111/jcmm.70079)
Supplement: Supplementary file 2 — Material S2. [file JCMM-28-e70079-s002.zip › 7.GSEA analysis/1.ARL11/KEGG_REGULATION_OF_AUTOPHAGY.html]

Details for gene set KEGG\_REGULATION\_OF\_AUTOPHAGY[GSEA]

|  || Dataset | ARL11.group.cls#h\_versus\_l |
| Phenotype | group.cls#h\_versus\_l |
| Upregulated in class | l |
| GeneSet | KEGG\_REGULATION\_OF\_AUTOPHAGY |
| Enrichment Score (ES) | -0.42088005 |
| Normalized Enrichment Score (NES) | -1.2651613 |
| Nominal p-value | 0.1733871 |
| FDR q-value | 1.0 |
| FWER p-Value | 0.968 |
Table: GSEA Results Summary

  

Fig 1: Enrichment plot: KEGG\_REGULATION\_OF\_AUTOPHAGY      
 Profile of the Running ES Score & Positions of GeneSet Members on the Rank Ordered List

  

| SYMBOL | TITLE | RANK IN GENE LIST | RANK METRIC SCORE | RUNNING ES | CORE ENRICHMENT || 1 | IFNA6 | na | 3473 | 0.220 | -0.0364 | No |
| 2 | IFNA14 | na | 4269 | 0.191 | -0.0041 | No |
| 3 | IFNA16 | na | 4914 | 0.169 | 0.0261 | No |
| 4 | IFNA4 | na | 6226 | 0.131 | 0.0262 | No |
| 5 | ULK2 | na | 8616 | 0.081 | -0.0193 | No |
| 6 | ATG4B | na | 8812 | 0.078 | -0.0024 | No |
| 7 | INS | na | 9462 | 0.068 | -0.0015 | No |
| 8 | PRKAA2 | na | 11235 | 0.045 | -0.0396 | No |
| 9 | ATG4D | na | 12449 | 0.032 | -0.0653 | No |
| 10 | BECN1 | na | 13580 | 0.022 | -0.0915 | No |
| 11 | ATG7 | na | 13648 | 0.022 | -0.0871 | No |
| 12 | PIK3R4 | na | 13845 | 0.020 | -0.0870 | No |
| 13 | IFNA2 | na | 15087 | 0.011 | -0.1197 | No |
| 14 | PRKAA1 | na | 17397 | -0.006 | -0.1845 | No |
| 15 | ULK1 | na | 19739 | -0.022 | -0.2456 | No |
| 16 | ULK3 | na | 21253 | -0.033 | -0.2796 | No |
| 17 | GABARAPL2 | na | 21878 | -0.038 | -0.2865 | No |
| 18 | GABARAP | na | 21909 | -0.039 | -0.2761 | No |
| 19 | ATG5 | na | 22396 | -0.044 | -0.2774 | No |
| 20 | ATG4A | na | 22729 | -0.047 | -0.2733 | No |
| 21 | PIK3C3 | na | 23449 | -0.055 | -0.2781 | No |
| 22 | ATG3 | na | 23824 | -0.060 | -0.2715 | No |
| 23 | GABARAPL1 | na | 24628 | -0.071 | -0.2741 | No |
| 24 | ATG4C | na | 24831 | -0.074 | -0.2585 | No |
| 25 | ATG12 | na | 27016 | -0.109 | -0.2901 | No |
| 26 | IFNA8 | na | 31555 | -0.242 | -0.3509 | Yes |
| 27 | IFNA10 | na | 33230 | -0.339 | -0.3011 | Yes |
| 28 | IFNA7 | na | 33254 | -0.341 | -0.2030 | Yes |
| 29 | IFNG | na | 33859 | -0.398 | -0.1053 | Yes |
| 30 | IFNA21 | na | 34187 | -0.451 | 0.0156 | Yes |
Table: GSEA details [plain text format]

  

Fig 2: KEGG\_REGULATION\_OF\_AUTOPHAGY      
 Blue-Pink O' Gram in the Space of the Analyzed GeneSet

  

Fig 3: KEGG\_REGULATION\_OF\_AUTOPHAGY: Random ES distribution      
 Gene set null distribution of ES for **KEGG\_REGULATION\_OF\_AUTOPHAGY**
